# Comparative genome-wide analysis of *CAD* (Cinnamyl Alcohol Dehydrogenase) gene family in *Medicago truncatula* and *Lotus japonicus* and their expression profiles in response to various abiotic abiotic stresses

**DOI:** 10.1371/journal.pone.0353726

**Published:** 2026-07-21

**Authors:** Mst. Sumaiya Khatun, Fatema Tuz Zohra, Pollob Shing, Md Shohel Ul Islam, Shaikh Mizanur Rahman, Md. Abdur Rauf Sarkar

**Affiliations:** 1 Department of Genetic Engineering and Biotechnology, Faculty of Biological Science and Technology, Jashore University of Science and Technology, Jashore, Bangladesh; 2 Department of Genetic Engineering and Biotechnology, Faculty of Biological Sciences, University of Rajshahi, Rajshahi, Bangladesh; Nuclear Science and Technology Research Institute, IRAN, ISLAMIC REPUBLIC OF

## Abstract

The CAD (Cinnamyl Alcohol Dehydrogenase) gene family is a key determinant for lignin biosynthesis in plants. In legumes, CAD enzymes are involved in the development of vascular tissues such as xylem and Casparian strip and they contribute to the production of antimicrobial and antifungal compounds. Thereby, it offers defense against pathogens and pests. Despite their biological significance, a comparative genome-wide analysis of the CAD gene family in *Medicago truncatula* and *Lotus japonicus* has not been explored. Therefore, we conducted a comparative genome-wide study to investigate the characteristics and potential role of CAD genes in these two model legume species. A total of 51 CAD genes were identified in *M. truncatula* (*MtCAD*) and 35 in *L. japonicus* (*LjCAD*). The CAD proteins are prominently characterized by ADH_N and ADH_zinc_N domains that were distributed in 8 and 6 chromosomes of *MtCAD* and *LjCAD*, respectively. Structural organization and conserved motif analysis indicated notable similarities between MtCAD and LjCAD proteins. However, considering the ancestry and functionality and based on the evolutionary analysis, *LjCAD* showed more similarities with *Arabidopsis* than *MjCAD*. Gene duplication analysis identified twelve duplicated gene pairs in *MtCAD* and eight in *LjCAD,* including both tandem and segmental duplication events*.* Most *MtCAD* and *LjCAD* were found in the cytoplasm with some of the *cis*-acting regulatory elements associated with stress responses. Gene Ontology annotation suggested that most *MtCAD* genes were associated with biological processes whereas *LjCAD* genes are mainly enriched in molecular functions. Both *MtCAD* and *LjCAD* showed potential roles in secondary metabolite production. Three substantial transcription factor families such as bZIP, C2H2, and ERF and several unique microRNAs were predicted to target *MtCAD* and *LjCAD* in regulating their gene expression against certain abiotic stressors for instance cold, freezing, drought, and heat. The *MtCAD* and *LjCAD* expressed highly in stress-responsive tissues such as nodule, root, immature flower, seed, and leaf. Meanwhile, RNA-sequencing data further highlighted several potential stress-responsive genes. In *M. truncatula*, The *MtCAD1*, *MtCAD3*, *MtCAD9*, *MtCAD15*, *MtCAD23*, *MtCAD27*, and *MtCAD47* exhibited higher expression under cold, drought, and freezing stress compared with control conditions. Whereas in *L. japonicus*, *LjCAD6*, *LjCAD8*, and *LjCAD11* showed higher expression under cold, drought, and heat stress. Thus, these genes may serve as promising candidates for improving abiotic stress tolerance and provide molecular insights into their functional roles for future crop improvement programs and experimental validation.

## 1. Introduction

The CAD (Cinnamyl Alcohol Dehydrogenase) gene family is a multigene family with tissue-specific or stress-inducible isoforms [1]. In plants, one of the most essential functions of the *CAD* gene is their role in lignin biosynthesis which modifies the plant’s structure, defense systems, and stress tolerance [2]. Upon pathogen recognition, signaling pathways activate defense-related gene expression, leading to the biosynthesis of phytoalexins, such as terpenoids and phenylpropanoids [3]. Phenylpropanoids are antimicrobial compounds that catalyse the final step in monolignol biosynthesis where cinnamyl aldehydes are converted into their corresponding alcohols by CAD enzymes [4]. During polymerisation, monolignols produce lignin which strengthens the secondary walls of stems and roots as well as vascular tissues [5]. The phenylpropanoid pathway produces monolignols like p-coumaryl, coniferyl, and sinapyl alcohols. The oxidative coupling of monolignols produces lignin as a complex polymer with hydroxyphenyl (H) guaiacyl (G) and syringyl (S) units [6]. The process of lignin synthesis shows variation because monolignols undergo different degrees of coupling depending on species, tissue type, and developmental stage [7]. In vascular tissues, particularly the xylem, lignin plays a crucial role in transporting water and provides structural support as well as resistance against pathogens and environmental stresses [8].

The CAD gene activity is associated with the conserved ADH_N and ADH_zinc_N domains. ADH_ N domain catalyzes cinnamyl aldehydes and maintains the overall structure and function of the enzyme [9]. Meanwhile, ADH_zinc_ N domain specifically binds two zinc ions-one for catalysis and one for structural stability and ensures proper enzyme function, substrate binding, and lignin polymerisation [10]. In response to pathogenic threats, plants strengthen their cell walls through lignification. For instance, *Verticillium dahliae* infection activates lignification in several plant species [11]. The roots of tomato show a rise in lignin synthesis after pathogen resistance, while the cotton plant shows *V. dahliae* elicitor-mediated lignin deposition through transcriptomic evidence of gene upregulation [12, 13]. Additionally, transcription factor GhMYB4 and the enzyme Gh4CL30 have been reported to regulate defense response through modification of cotton lignification processes [14].

*Medicago truncatula* and *Lotus japonicus* were selected for comparative analysis because they are well-established model legumes with complementary ecological and physiological traits. Although, both species share conserved nodulation and lignin biosynthesis pathways, they differ in stress responses and environmental adaptation. *M. truncatula*, a forage legume, was first introduced as a model legume in 1990 [15]. Meanwhile, *L. japonicus* was introduced as a model legume in 1992 [16]. Since then, both the species have contributed significantly to the advancements of legume biology. They have been widely used to study rhizobia Nod factor recognition and the signaling networks regulating nodule formation [17]. They have also been facilitated the identification of genetic mechanisms that enhance tolerance to salinity and drought conditions. For instance, the activation of *CCaMK* and a gain of function mutation in *LHK1* can induce this process without requiring rhizobial signals [18].

Despite their shared characteristics as model legumes, *M. truncatula* and *L. japonicus* differ in their ecological adaptation and stress response mechanisms. *M. truncatula* is primarily adapted to Mediterranean environments and has been widely used to study drought and salinity tolerance [19]. In contrast, *L. japonicas*, native to temperate East Asia, thrives across varied environments and exhibits distinct symbiotic relationships and stress response mechanisms [20].

The comparative genome-wide study on *MtCAD* and *LjCAD* genes provides insights into the evolutionary, structural, and functional characteristics in of the CAD gene family in *M. truncatula* and *L. japonicus*. The evolutionary phylogenetic tree analysis revealed more genetic resemblance of LjCAD with AtCAD than MtCAD. Gene structure, conserved domain, and motif analysis further demonstrated functional and structural conformity among different CAD subgroups. Gene duplication analysis indicated that the evolution of MtCAD and LjCAD genes was primarily driven by purifying selection, with both tandem and segmental duplication events contributing to their expansion. GO and KEGG pathway analyses further clarified their biological roles and metabolic functions, while CARE analysis revealed their involvement particularly with presence of multiple the stress responsive signaling motif. Additionally, tissue-specific expression patterns and RNA-sequencing data under various abiotic stresses further supported these findings. Based on the role of CAD genes in lignin biosynthesis and plant defense system, we hypothesize that the expansion and diversification of the CAD gene family in *M. truncatula* and *L. japonicus* contribute to functional specialization in abiotic stress adaptation. We further hypothesize that specific CAD gene clades possess stress-responsive regulatory elements and display differential expression patterns under various abiotic stress conditions.

## 2. Methods and materials

### 2.1. Identification and characterization of CAD genes in legume families (*Medicago* and *Lotus*)

The CAD gene DNA binding domains from *Arabidopsis* were used as queries to retrieve CAD-related gene sequences, whole genome sequences, and protein sequences from *Medicago truncatula* and *Lotus japonicas* (S1–S5 Data in S1 File). These sequences were obtained from Phytozome v13 using BLASTp (Protein-Basic Local Alignment Search Tool), with an E-value threshold set to −1, a comparison matrix of BLOSUM62, and default parameters [21]. To identify conserved domain patterns across CAD protein sequences, several tools were employed, including SMART Simple Modular Architecture Research Tool (SMART) [22], NCBI CDD (Conserved Domain Database) [23], and PfamScan [24], all using default settings. The candidate were selected based on the presence of ADH_N domain.

### 2.2. Determination of physiochemical properties of MtCAD and LjCAD

The physicochemical properties of MtCAD and LjCAD were analyzed, including the number of amino acid residues, molecular weight (in kDa), isoelectric point (pI), instability index, aliphatic index, and Grand Average of Hydropathy (GRAVY). These properties were determined using the ProtParam online tool available at

### 2.3. Phylogenetic analysis among MtCAD, LjCAD, AtCAD, OsCAD, SbCAD, HvCAD, and BdCAD

Phylogenetic tree analysis was performed using MEGA11 software [25]. The tree was constructed using the Maximum Likelihood (ML) model [26] with Jones–Taylor–Thornton (JTT) as substitution model. Uniform rates among sites and Nearest-Neighbor-Interchange (NNI) heuristic search were applied. Meanwhile, a total of 1000 bootstrap replicates were conducted to evaluate branch support. The ClustalW program was utilized for sequence alignment [27]. The resulting phylogenetic tree was visualized using the ChiPlot online tool [28].

### 2.4. Conserved motif, domain, and gene structure analysis of *MtCAD* and *LjCAD*

Conserved motif analysis was performed using the MEME (Multiple EM for Motif Elicitation) suite [29] with default parameters except the motif parameter set to 10. Visualizations were made using the ChiPlot online program. Domain organization was identified using the Motif Search tool [30] and further visualized using DOG2.0 software [31]. Gene structure analysis, including exon-intron organization, was performed using the Gene Structure Display Server (GSDS) version 2.0 [32].

### 2.5. Time divergence, gene duplication, and Ka/Ks ratio calculation of *MtCAD* and *LjCAD*

The Ka/Ks ratios and divergence times of duplicated gene pairs in MtCAD and LjCAD were calculated using TBtools v2.154 [33]. The divergence time between *MtCAD* and *LjCAD* was calculated in million years ago (MYA) using T = Ks/2λ, where λ represents the neutral substitution rate (6.5 × 10^−9^ substitutions per site per year) [34].

### 2.6. Collinearity, synteny and chromosomal localization analysis of *MtCAD* and *LjCAD*

Collinearity and synteny relationships among CAD genes were analyzed using TBtools v2.154. The MCScanX algorithm was used to process the whole genome sequences and genome annotation files [35]. The MG2C web v2 server mapped and displayed the chromosomal locations of *MtCAD* and *LjCAD* [36].

### 2.7. Prediction of subcellular localization and *cis*-acting regulatory elements in *MtCAD* and *LjCAD*

The sub cellular localizations were predicted using the Wolf PSORT online program predicted protein sub cellular localization [37]. Sequences of 5′ UTR spanning 2000 base pairs were extracted for both *MtCAD* and *LjCAD* from the plant CARE database. [38]. The graphical representation was performed in RStudio version 2024.09.0 [39].

### 2.8. Gene ontology (GO), protein-protein interactions (PPIs), KEGG pathway (Kyoto Encyclopedia of Genes and Genomes) analysis of *MtCAD* and *LjCAD*

Gene Ontology (GO) annotation was predicted using the PlantRegMap tool with a threshold p-value of 0.01 and default parameters [40]. Protein-protein interactions and KEGG pathway analysis were performed using the STRING version 12 online tool by utilizing peptide sequences (S3 Data in S1 File) [41]. The visualization of GO functions and KEGG pathways was performed using SRPLOT [42].

### 2.9. Transcription factors (TFs) and regulatory network analysis of *MtCAD* and *LjCAD*

Information on transcription factors potentially regulating MtCAD and LjCAD genes was obtained from the PlantTFDB database using default parameters and a threshold p-value of 1 × 10 ⁻ ⁴. Regulatory networks between CAD genes and transcription factors were constructed and visualized using Cytoscape software version 3.10.3 [43].

### 2.10. Prediction of putative micro-RNAs (miRNAs) targeting *MtCAD* and *LjCAD*

Putative microRNAs (miRNAs) targeting *MtCAD* and *LjCAD* genes were predicted using the miRBase database [44] and the psRNATarget server [45]. The miRNA-gene regulatory networks were visualized using Cytoscape version 3.10.3.

### 2.11. Tissue-specific and various abiotic stress treatment expression pattern in *MtCAD* and *LjCAD*

The tissue-specific expression data of *MtCAD* genes (bio project ID: PRJNA80163) [46] were obtained from NCBI Sequence Read Archive (SRA) and the *LjCAD* genes tissue-specific expression data were retrieved from Lotus japonicus Expression Atlas [47]. The RNA-sequencing data of various abiotic stressors treatment were generated with bio project ID: PRJNA884427 for *LjCAD* [48] and bio project ID: PRJNA286829 for *MtCAD* from NCBI [49]. For quality control and trimming, version 0.32 of trimmomatic package was used [50]. Clean reads were aligned to the reference genomes of *M. truncatula* and *L. japonicus* using STAR v2.7.11b [51]. Samtools packages version 1.20 was used for the conversion of the sequence alignment map (SAM) to binary alignment map (BAM) files [52]. Gene expression levels were quantified as Fragments Per Kilobase of transcript per Million mapped reads (FPKM) using RSEM v1.1.17 [53]. The expression patterns were visualized using TB tools v2.154.

## 3. Results

### 3.1. Identification and determination of physiochemical properties of MtCAD and LjCAD

A comprehensive analysis identified a total of 86 cinnamyl alcohol dehydrogenase (CAD) genes, comprising 51 *MtCAD* in *M. truncatula* and 35 *LjCAD* in *L. japonicus*. The length of amino acid in *M. truncatula* ranged from 109 A.A. (MtCAD51) to 633 A.A. (MtCAD16 & MtCAD17) (Table 1). Meanwhile, the length of amino acid in *L. japonicus* ranged from 312 A.A. (LjCAD15) to 671 A.A. (LjCAD21). Accordingly, LjCAD proteins were generally heavier ranging from 33.29 kDa (LjCAD15) to 70.77 kDa (LjCAD21). In contrast, the molecular weight of MtCAD ranged from 11.43 kDa (MtCAD51) to 68.89 kDa (MtCAD17). Both MtCAD and LjCAD showed higher number of acidic proteins. For example, 29 MtCAD were acidic while 22 LjCAD were acidic. Further, 39 MtCAD had instability index lower than 40, whereas 31 LjCAD proteins had instability index below 40. Two protein groups displayed predominantly hydrophobic characteristics in their GRAVY value yet MtCAD exhibited a broader hydrophobicity scale due to MtCAD51 (0.304) being its most hydrophobic member alongside MtCAD29 (−0.242). Meanwhile, LjCAD comprised most hydrophilic LjCAD1 (0.242) and most hydrophobic LjCAD4 (−0.163).

**Table 1:** Physiochemical properties of MtCAD and LjCAD proteins.

Sequence ID	Number of Amino Acid	Molecular Weight	Theoretical pI	Instability Index	Aliphatic Index	Grand Average of Hydropathicity
*MtCAD1*	360	38982.79	6.22	28.25	92.89	−0.001
*MtCAD2*	379	40463.50	6.07	30.72	88.71	0.104
*MtCAD3*	358	38954.93	5.55	26.53	94.05	0.005
*MtCAD4*	406	44320.49	6.67	27.65	84.66	−0.091
*MtCAD5*	325	34554.79	6.90	39.75	95.97	0.092
*MtCAD6*	325	34746.09	6.91	42.09	97.82	0.054
*MtCAD7*	370	40266.39	8.83	40.01	90.30	−0.119
*MtCAD8*	386	41701.23	6.03	39.49	86.58	0.065
*MtCAD9*	380	41097.26	5.97	27.59	84.89	−0.037
*MtCAD10*	381	41205.33	5.76	30.53	85.43	−0.006
*MtCAD11*	380	41108.25	5.97	26.62	85.42	−0.036
*MtCAD12*	346	37178.42	9.12	20.84	115.20	0.266
*MtCAD13*	391	42503.08	6.28	30.01	92.17	−0.037
*MtCAD14*	374	39908.92	8.24	37.83	95.86	0.055
*MtCAD15*	325	34554.79	6.90	39.75	95.97	0.092
*MtCAD16*	633	68612.63	9.03	21.32	92.43	0.038
*MtCAD17*	633	68895.24	9.15	24.09	96.24	0.076
*MtCAD18*	361	39124.22	7.15	28.11	87.20	−0.036
*MtCAD19*	361	38829.14	6.70	25.67	90.17	0.066
*MtCAD20*	361	38855.01	7.15	26.95	83.07	−0.002
*MtCAD21*	361	38773.75	6.46	26.07	96.07	0.061
*MtCAD22*	361	38939.92	6.21	27.69	95.54	0.042
*MtCAD23*	361	39044.92	5.95	27.51	87.23	−0.096
*MtCAD24*	194	21421.84	6.70	22.96	88.40	−0.139
*MtCAD25*	362	39108.18	6.42	22.80	93.92	0.035
*MtCAD26*	362	39002.90	5.67	22.05	88.81	0.006
*MtCAD27*	362	38935.84	5.72	23.29	89.89	0.014
*MtCAD28*	363	39039.17	7.62	26.45	90.17	0.036
*MtCAD29*	110	12566.43	8.42	39.38	78.73	−0.242
*MtCAD30*	361	39167.18	6.50	25.74	93.16	−0.045
*MtCAD31*	358	38560.60	6.66	35.33	87.40	0.009
*MtCAD32*	374	40067.44	6.60	32.41	91.42	0.147
*MtCAD33*	391	42736.76	5.56	35.54	91.00	0.109
*MtCAD34*	309	33274.14	5.38	30.30	99.35	−0.004
*MtCAD35*	320	34505.58	5.57	31.48	101.44	−0.008
*MtCAD36*	377	40536.93	7.46	38.39	89.39	0.086
*MtCAD37*	362	39002.32	6.13	32.05	93.92	0.117
*MtCAD38*	362	39002.32	6.13	32.05	93.92	0.117
*MtCAD39*	369	39822.61	8.98	32.77	97.48	0.078
*MtCAD40*	393	42415.75	5.86	28.25	85.57	−0.061
*MtCAD41*	385	41683.22	6.17	34.78	92.18	0.009
*MtCAD42*	380	40718.73	6.14	25.87	86.16	0.017
*MtCAD43*	342	37905.88	5.94	30.69	86.32	−0.109
*MtCAD44*	339	37448.38	6.27	27.98	89.68	−0.085
*MtCAD45*	384	40940.85	7.64	28.03	91.59	−0.026
*MtCAD46*	360	38652.87	6.16	26.28	99.31	0.108
*MtCAD47*	416	45803.94	8.94	30.49	88.97	−0.100
*MtCAD48*	425	45587.44	8.16	41.74	91.11	−0.037
*MtCAD49*	461	50357.40	6.88	36.84	95.77	0.068
*MtCAD50*	351	36803.37	5.55	33.04	101.48	0.274
*MtCAD51*	109	11433.20	4.94	18.78	116.33	0.304
*LjCAD1*	346	37210.46	8.94	19.21	114.08	0.242
*LjCAD2*	380	40851.93	6.38	30.83	84.58	−0.014
*LjCAD3*	650	69833.54	5.68	24.68	96.60	0.023
*LjCAD4*	338	36913.18	6.09	29.63	80.71	−0.163
*LjCAD5*	357	38864.45	5.71	29.08	83.25	−0.069
*LjCAD6*	380	41110.15	5.92	29.03	83.34	−0.021
*LjCAD7*	334	35265.07	9.37	31.32	96.65	0.012
*LjCAD8*	400	43731.11	6.07	28.28	81.60	−0.094
*LjCAD9*	380	41015.23	7.50	27.85	81.79	−0.023
*LjCAD10*	385	40990.58	5.88	34.30	88.88	0.158
*LjCAD11*	363	39169.61	7.10	29.43	92.87	0.056
*LjCAD12*	329	34734.51	9.05	33.71	102.31	0.108
*LjCAD13*	364	39233.68	6.31	29.84	91.87	0.104
*LjCAD14*	363	39235.27	5.91	27.11	97.74	0.043
*LjCAD15*	312	33289.17	5.27	32.53	99.68	0.029
*LjCAD16*	319	34154.35	5.91	29.60	99.06	0.012
*LjCAD17*	378	40587.85	6.32	40.09	86.30	0.060
*LjCAD18*	330	34982.42	9.04	35.95	92.82	−0.014
*LjCAD19*	329	36278.45	5.21	30.58	88.57	−0.159
*LjCAD20*	377	40638.21	9.08	36.21	98.73	0.038
*LjCAD21*	671	70771.40	5.64	31.49	90.15	0.118
*LjCAD22*	410	44254.05	7.16	30.97	86.27	−0.055
*LjCAD23*	359	38615.41	6.31	26.88	93.37	0.021
*LjCAD24*	430	45644.52	8.39	42.54	90.98	0.066
*LjCAD25*	356	38924.44	6.00	30.80	82.92	−0.067
*LjCAD26*	633	68469.49	9.05	26.07	95.62	0.088
*LjCAD27*	387	41422.43	8.36	30.25	92.92	−0.018
*LjCAD28*	359	39108.72	6.26	30.01	88.77	−0.090
*LjCAD29*	379	40601.69	6.42	29.97	87.39	0.050
*LjCAD30*	358	39145.21	5.44	28.92	94.92	0.012
*LjCAD31*	355	38336.48	7.45	34.26	83.41	0.087
*LjCAD32*	355	38173.71	6.11	27.81	88.06	0.093
*LjCAD33*	389	42016.59	6.23	36.11	90.41	0.067
*LjCAD34*	325	34728.91	6.67	44.91	95.38	0.007
*LjCAD35*	428	46665.22	6.33	38.73	86.73	0.064

### 3.2. Phylogenetic relationship analysis among MtCAD, LjCAD, AtCAD, OsCAD, SbCAD, HvCAD and BdCAD

**Fig 1:**
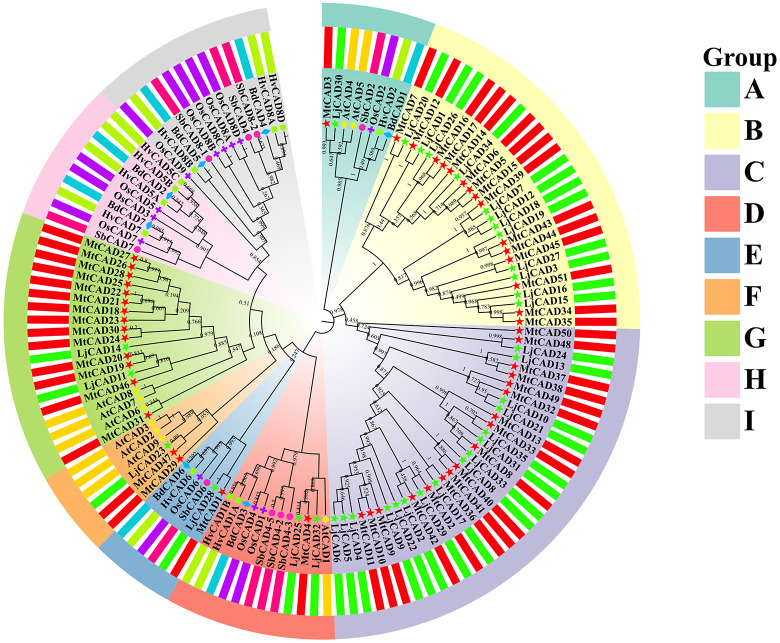
Phylogenetic relationship analysis among MtCAD, LjCAD, AtCAD, OsCAD, SbCAD, HvCAD and BdCAD. Phylogenetic relationship among MtCAD, LjCAD, AtCAD, OsCAD, SbCAD, HvCAD and BdCAD were classified into 7 groups (A-I). Each group shows distinct colors and shapes. The candidate MtCAD and LjCAD was labeled with red and green star, respectively. Whereas AtCAD was labeled as parallelogram with orange color, SbCAD with magenta circle, OsCAD with purple plus HvCAD with green rectangle and BdCAD with blue diamond.

A comparative phylogenetic tree was constructed by using a total of 134 CAD proteins, 51 from *M. truncatula*, 35 from *L. japonicus*, 9 from *A. thaliana*, 11 from *Hordeum vulgare*, 12 from *Oryza sativa*, 7 *Brachypodium distachyon* and 9 from *Sorghum bicolor* (Fig 1). Based on the distribution pattern of AtCAD proteins, the MtCAD and LjCAD genes were classified into nine groups (A–I). Among these, group C contained the highest number of members (33), including 17 MtCAD and 16 LjCAD genes. In contrast, groups E and F contained the fewest members (6 each). The remaining groups, A, B, D, G, H and I, contained 8, 28, 12, 16, 10, and 12 members, respectively (S1 Fig). Notably, this distribution pattern showed that LjCAD shared a closer evolutionary relationship with AtCAD.

### 3.3. Conserved motif, domain, and gene structure analysis of MtCAD and LjCAD

**Fig 2:**
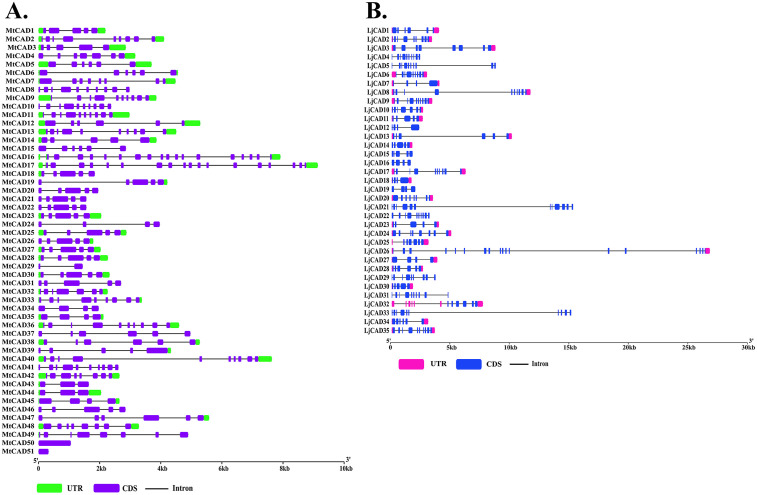
Gene structure analysis of *MtCAD* and *LjCAD.* A. MtCAD chromosome is shown in purple color. B. LjCAD chromosome is shown in light green color. The chromosome number is at the top of each chromosome bar. The millions of bases (Mb) indicate the length of each chromosome on the left.

In this study, 10 highly conserved motifs were analyzed for both *MtCAD* and *LjCAD*. The highest number of motifs (9) were found in both *MtCAD* and *LjCAD* (*MtCAD1*, *MtCAD3*, *MtCAD4*, *MtCAD18*, *MtCAD19*, *MtCAD20*, *MtCAD21*, *MtCAD22*, *MtCAD23*, *MtCAD25*, *MtCAD26*, *MtCAD27*, *MtCAD28*, *MtCAD30*, *MtCAD31*, *MtCAD46*, *MtCAD47*, *LjCAD2*, *LjCAD6*, *LjCAD8*, *LjCAD9*, *LjCAD10*, *LjCAD17*, *LjCAD22*, *LjCAD29*, *LjCAD33*, and *LjCAD35*). On the other hand, *MtCAD51* contains the lowest number of motif (1) (S2 Fig A). Meanwhile, the minimum number of motif was present in *LjCAD19* (2) (S2 Fig B). These conserved regions are critical for maintaining the catalytic of CAD proteins. Notably, the conserved domain analysis identified ADH_N domain along with ADH_zinc_ N domain (except *MtCAD29* and *MtCAD51*) that were present in both *MtCAD* and *LjCAD* (S3 Fig A and B). Accordingly, the gene structure analysis revealed the presence of intron in 49 *MtCAD* genes (except *MtCAD50* and *MtCAD51*) (Fig 2 A). Whereas, all the *LjCAD* genes contained intron (Fig 2 B). The highest number of introns in *MtCAD* and *LjCAD* were 18 and 17, respectively (S6 Data in S1 File).

### 3.4. Chromosomal localization, collinearity, and synteny analysis of *MtCAD* and *LjCAD*

The 51 *MtCAD* were distributed across 8 chromosomes (S4 Fig A), while the 35 *LjCAD* genes were distributed on 6 chromosomes (S4 Fig B). This distribution showed that the highest number of *MtCAD* (15) were located on chromosome 5. Meanwhile, the highest number of *LjCAD* (10) were present on chromosome 1. Notably, three scaffold chromosome in *MtCAD*s (*MtCAD49*, *MtCAD50* and *MtCAD51*) and 1 contig chromosome in *LjCAD* (*LjCAD35*) were found.

Additionally, 12 collinear pairs of *MtCAD* and 8 collinear pairs of *LjCAD* were identified in this analysis. In *M. truncatula*, chromosomes 3, 5, and 7 participated most frequently in forming collinear gene pairs with other chromosomes (S5 Fig A). Chromosome 1 and chromosome 2 also formed collinear pairings more frequently compared to the remaining chromosomes. For example, *MtCAD2* on chromosome 1 paired with *MtCAD42* on chromosome 7*, MtCAD8* on chromosome 3 paired with *MtCAD33* on chromosome 5, and *MtCAD3* on chromosome 1 paired with *MtCAD29* on chromosome 5. On the other hand, *LjCAD10* on chromosome 1 paired with *LjCAD21* on chromosome 3, *LjCAD12* on chromosome 2 paired with *LjCAD21* on chromosome 3, and *LjCAD25* on chromosome 4 paired with *LjCAD32* on chromosome 6 (S5 Fig B).

**Fig 3:**
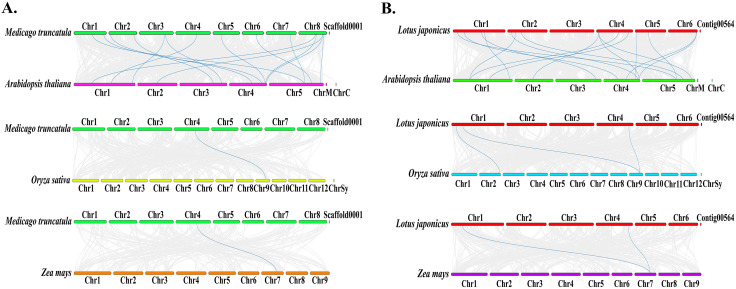
Synteny analysis of *MtCAD* and *LjCAD.* A. The synteny of M. truncatula with A. thaliana, O. sativa, and Z. mays. The green, magenta, yellow, and orange rectangles represent M. truncatula, A. thaliana, O. sativa, and Z. mays, respectively. B. The synteny of L. japonicas with A. thaliana, O. sativa, and Z. mays. The red, green, sky blue, and purple rectangles represent L. japonicas, A. thaliana, O. sativa, and Z. mays, respectively. The blue color line indicates a syntenic relationship between the species.

The syntenic analysis further revealed that *MtCAD* genes formed 15 pairs with *A. thaliana*, 1 pair with *O. sativa,* and 1 pair with *Z. mays* (Fig 3 A)*.* Meanwhile, *LjCAD* had 16 pairs with *A. thaliana*, 2 pairs with *O. sativa*, and 2 pairs with *Z. mays* (Fig 3 B). The pairing concluded that *LjCAD* had a closer relationship with *Arabidopsis*.

### 3.5. Gene duplication, time divergence, and Ka/Ks ratio calculation of *MtCAD* and *LjCAD*

In this analysis, 12 pairs of duplicated genes were found in *MtCAD*, while 8 pairs of duplicated genes were found in *LjCAD* (S7 Data in S1 File). Additionally, 4 segmental and 6 tandem duplications were exhibited in *MtCAD*. Whereas, 5 segmental and 3 tandem duplications were found in *LjCAD*. In *MtCAD*, the evolutionary time divergence varied from 6.080145846 MYA (*MtCAD43-MtCAD44*) to 77.80829779 MYA (*MtCAD3-MtCAD29*) (S6 Fig A). In LjCAD, it ranged from 8.029178047 MYA (*LjCAD5-LjCAD6*) to 48.70931528 MYA (*LjCAD11-LjCAD14*) (S6 Fig B). The Ka/Ks ratios of all duplicated gene pairs were less than 1 in both the species. Thus, it indicated that these genes have predominantly evolved under purifying selection. This further suggested that selective pressure has acted to maintain the functional conservation of duplicated CAD genes during evolution.

### 3.6. Prediction of subcellular localization and *cis*-acting regulatory elements (CARE) in *MtCAD* and *LjCAD*

Subcellular localization prediction suggested that MtCAD proteins might distribute across several cellular compartments including the nucleus, mitochondria, cytoplasm, chloroplast, peroxisome, cytoskeleton, Golgi apparatus, vacuole, endoplasmic reticulum, plasma membrane, and extracellular space (S7 Fig A). However, the majority of MtCAD proteins (92.16%) were predicted to localize in the cytoplasm (S7 Fig B). Similarly, most LjCAD proteins (85.71%) were predicted to be cytoplasmic (S7 Fig C) while a smaller proportion showed potential localization signals for other compartments, including the chloroplast (S7 Fig D). These results suggested that cytoplasmic localization is the predominant prediction for CAD proteins in both species whereas other predicted localizations might represent secondary or lower-confidence signals.

**Fig 4:**
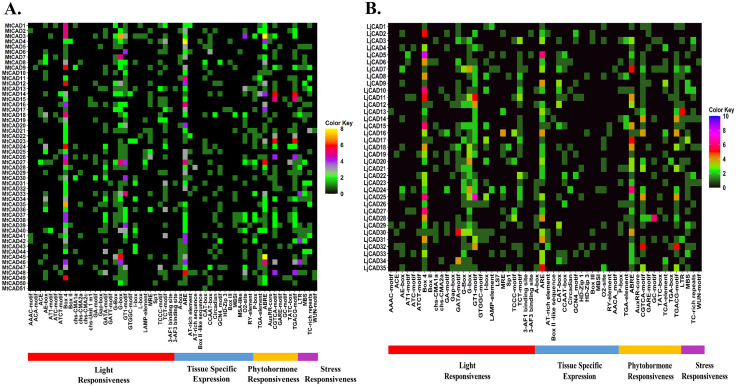
Prediction of *cis*-acting regulatory elements (CARE) in *MtCAD* and *LjCAD.* A. Color bar of MtCAD CARE (black = 0, green = 1–2, blue = 2-4, red = 4-6, and yellow = 6-8) is illustrated. B. Different colors (black = 0, green = 1–2, yellow = 2-4, red = 4-6, magenta = 6-8, blue = 8-10) of LjCAD is illustrated, Functions associated with CAREs are shown at the bottom of the heat map.

CARE analysis included 60 motifs of *MtCAD* and 56 motifs of *LjCAD that* were classified into 4 groups, such as light responsiveness (LR), tissue-specific expression, phytohormone responsiveness (PR), and stress responsiveness (SR) (S8 Data in S1 File). The majority of *MtCAD* and *LjCAD* motifs were associated with light responsiveness. In both *MtCAD* and *LjCAD,* the Box 4 motif was present predominantly (Fig 4 A and B). Several stress and hormone responsive elements were also widely distributed including ARE and ABRE motifs which are commonly associated with anaerobic and abscisic acid-mediated stress responses. In addition, TGACG-motif and CGTCA-motif (MeJA-responsive elements) as well as GT1-motif, G-box, and WUN-motif were identified in multiple CAD promoters. This suggested potential regulatory roles in stress signaling and defense responses.

### 3.7. Gene Ontology (GO), Protein-Protein Interaction (PPIs), KEGG (Kyoto Encyclopedia of Genes and Genomes) pathway analysis of *MtCAD* and *LjCAD*

**Fig 5:**
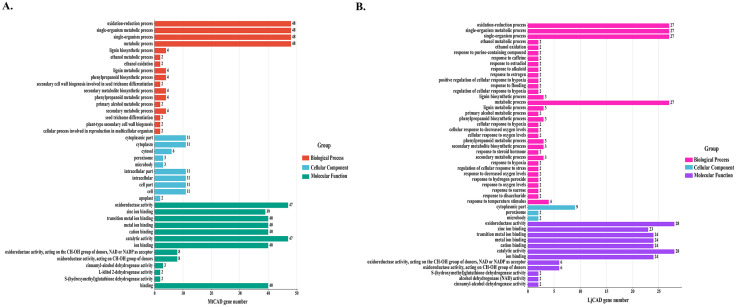
Gene Ontology (GO) pathway analysis of *MtCAD* and *LjCAD.* A. Different color bar represents different function. The higher bar, the more the MtCAD genes are present. B. Different color bar represents different function. The higher bar, the more the LjCAD genes are present.

In the gene ontology, 40 and 48 GO annotations were identified in *MtCAD* and *LjCAD* grouped into 3 categories: biological process (BP), cellular component (CC), and molecular function (MF) (S9 Data in S1 File). In *MtCAD*, most genes were associated with biological processes related to oxidation-reduction reactions and metabolic processes (Fig 5 A). In contrast, *LjCAD* genes were mainly enriched in molecular functions such as oxidoreductase activity and ion binding (Fig 5 B).

The PPI showed that MtCAD18, MtCAD20, MtCAD21, MtCAD22, MtCAD25, MtCAD26, MtCAD27, MtCAD28, MtCAD30, and MtCAD31 exhibited higher interactions with CAD7 (S8 Fig A). Furthermore, CAD7 interacted with CAD1–2, PDC3, PER48, CAD8, PDC4, and SDH (S10 Data in S1 File). Meanwhile, LjCAD showed strong interactions with ADH1, which, in turn, interacted with F2K13.90 and PDC3 (S8 Fig B).

**Fig 6:**
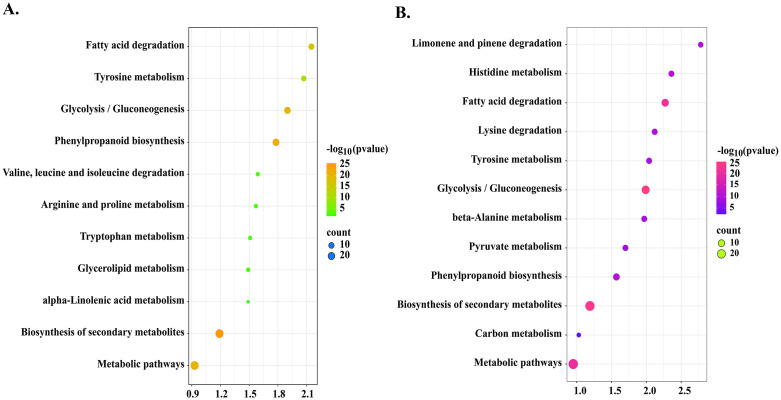
KEGG (Kyoto Encyclopedia of Genes and Genomes) pathway analysis of *MtCAD* and *LjCAD.* A. The metabolic pathway of the MtCAD. The p-value range and number of gene count in small to large round are represented in the right side. B. The metabolic pathway of the LjCAD. The p-value range and number of gene count in small to large round are represented in the right side.

KEGG pathway mapping highlighted that both *MtCAD* and *LjCAD* genes were involved in several metabolic pathways, including fatty acid degradation, tyrosine metabolism, glycolysis/gluconeogenesis, phenylpropanoid biosynthesis, and the biosynthesis of secondary metabolites (S11 Data). Among these, the phenylpropanoid biosynthesis pathway is particularly relevant because it included key reactions involved in lignin production. Some pathways are species specific as *MtCADs* were associated with valine, leucine, and isoleucine degradation, arginine and proline metabolism, tryptophan metabolism, glycerolipid metabolism, and alpha-linolenic acid metabolism (Fig 6 A) while *LjCADs* participated in limonene and pinene degradation, histidine metabolism, lysine degradation, beta-alanine metabolism, pyruvate metabolism, and carbon metabolism (Fig 6 B).

### 3.8. Transcription factors and regulatory network analysis of MtCAD and LjCAD

In MtCAD and LjCAD*,* a total of 103 and 102 unique TFs were predicted correspondingly. About 7 TFs were predominantly found in both species*.* In MtCAD, the major TF families included ERF, MYB, bZIP, NAC, LBD, GATA, and Nin-like (S9 Fig A), whereas ERF, NAC, MYB, bHLH, MIKC_MADS, LBD, and GATA were identified in LjCAD (S9 Fig B)*.* The largest family found in both MtCAD and LjCAD was ERF. For example, ERF members such as Medtr5g008590, Medtr8g092460, Medtr3g072610, Medtr5g083340, and Medtr2g015050 were associated with multiple MtCAD genes, while Lj6g3v0841450, Lj6g3v2007040, Lj2g3v2113110, Lj6g3v2006220, Lj0g3v0114779, and Lj2g3v1989210 were predicted to interact with several LjCAD. The ERF family showed the highest number of predicted interactions, potentially regulating 36 MtCAD and 28 LjCAD genes (S10 Fig A and B).

### 3.9. Prediction of putative micro-RNAs targeting *MtCAD* and *LjCAD*

A total of 510 and 21 mature miRNAs were predicted to target 49 MtCAD and 12 LjCAD genes, respectively (S12 Data in S1 File). Among them, mtr-miR2086-5p targeted the largest number of *MtCAD* genes, including *MtCAD9*, *MtCAD10*, *MtCAD11*, *MtCAD16*, *MtCAD19*, *MtCAD20*, *MtCAD32*, *MtCAD43*, and *MtCAD44* (S8 Fig A). Some miRNA such as mtr-miR169c targeted *MtCAD21, MtCAD22, MtCAD26, MtCAD27, MtCAD28, MtCAD31, MtCAD43*, and *MtCAD44* and mtr-miR2592a-3p also targeted *MtCAD30, MtCAD34, MtCAD35, MtCAD9, MtCAD10*, and *MtCAD50*). Likewise, in *LjCAD,* lja-miR7533a targeted *LjCAD15* and *LjCAD16*, while the rest of the miRNAs targeted one specific *LjCAD* (S11 Fig B). These predicted interactions suggest possible post-transcriptional regulation of CAD genes by miRNAs in both species.

### 3.10. Tissue-specific and various abiotic stress treatment expression in *MtCAD* and *LjCAD*

**Fig 7:**
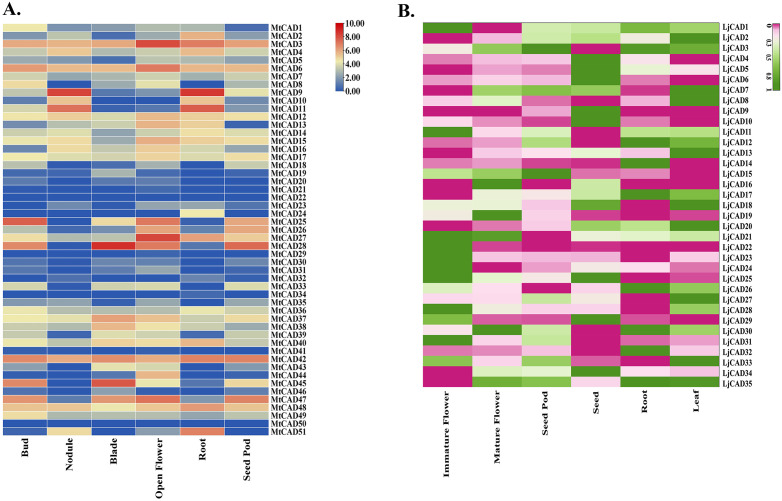
Tissue-specific expression in *MtCAD* and *LjCAD.* A. A heat map representing the tissue expression pattern in MtCAD is shown. B. A heat map representing the tissue expression pattern in LjCAD is shown. Various tissues are at the bottom of the heat map. The color bar represents the intensity of the expression.

In this analysis, the expression of *MtCAD* in different tissues such as bud, nodule, blade, open flower, root, and seed pod, while the expression of *LjCAD* in immature flower, mature flower, seed pod, seed, root, and leaf had been studied (S13 Data in S1 File, Fig 7 A and B). For each gene, the tissue showing the highest observed expression was identified. In *MtCAD*, *MtCAD25* exhibited the highest expression in buds, *MtCAD27* in open flowers, and *MtCAD28* in blades. Root-associated expression was observed for *MtCAD3*, *MtCAD6*, and *MtCAD9*, which showed their highest expression in roots and nodules. In *LjCAD*, the highest observed expression was detected in immature flowers for *LjCAD23* and in mature flowers, seeds, and roots for several other genes, including *LjCAD30* and *LjCAD32*. These observations indicate the tissues where CAD genes might be preferentially expressed. These results represent indicative tissue-specific expression trends.

**Fig 8:**
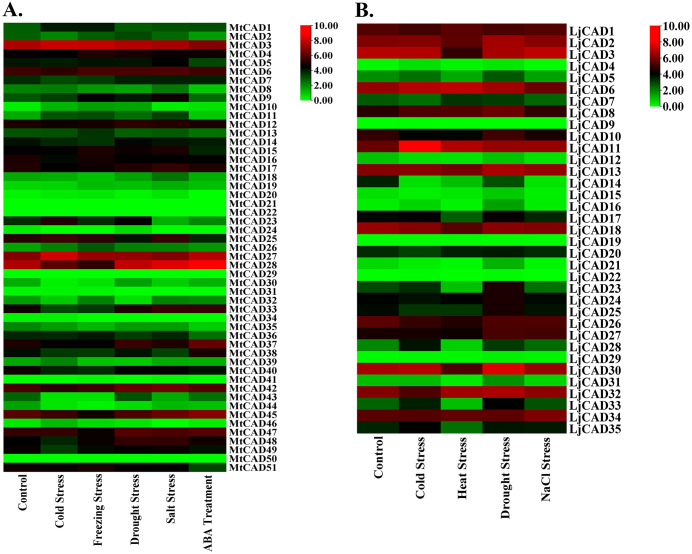
Various abiotic stress treatment expression in *MtCAD* and *LjCAD.* A. A heat map representing different stressed expression in MtCAD is shown. B. A heat map representing different stressed expression in LjCAD is shown. The bottom of the heat map contains levels of treatment and color bar represents the intensity of expression.

The expression in various abiotic stressors were carried out to the expression pattern in both *MtCAD* and *LjCAD* (S14 Data in S1 File, Fig 8 A and B). In MtCAD, cold stress induced higher transcript levels in *MtCAD1*, *MtCAD3*, *MtCAD8*, *MtCAD9*, *MtCAD10*, *MtCAD11*, *MtCAD13*, *MtCAD15*, *MtCAD19*, *MtCAD20*, *MtCAD23*, *MtCAD25*, *MtCAD26*, *MtCAD27*, *MtCAD31*, *MtCAD34*, *MtCAD39*, *MtCAD46* and *MtCAD47* suggested their role as cold-responsive candidate genes pending functional validation. Some genes also exhibited highly during freezing stress such as, *MtCAD1*, *MtCAD2*, *MtCAD3*, *MtCAD4*, *MtCAD5*, *MtCAD6*, *MtCAD9*, *MtCAD10*, *MtCAD11*, *MtCAD13*, *MtCAD15*, *MtCAD19*, *MtCAD20*, *MtCAD23*, *MtCAD25*, *MtCAD26*, *MtCAD27*, *MtCAD32*, *MtCAD36*, *MtCAD41*, *MtCAD46*, *MtCAD48*, and *MtCAD51*. During drought stress, a subset of *MtCAD* genes, including *MtCAD1*, *MtCAD2*, *MtCAD3*, *MtCAD4*, *MtCAD5*, *MtCAD6*, *MtCAD7*, *MtCAD9*, *MtCAD10*, *MtCAD11*, *MtCAD12*, *MtCAD14*, *MtCAD15*, *MtCAD17*, *MtCAD18*, *MtCAD19*, *MtCAD23*, *MtCAD24*, *MtCAD26*, *MtCAD27*, *MtCAD28*, *MtCAD30*, *MtCAD33*, *MtCAD37*, *MtCAD42*, *MtCAD43*, *MtCAD46*, *MtCAD47*, and *MtCAD48* showed higher transcript levels compared to control. In salt stress, *MtCAD1*, *MtCAD4*, *MtCAD5*, *MtCAD6*, *MtCAD7*, *MtCAD8*, *MtCAD9*, *MtCAD10*, *MtCAD11*, *MtCAD12*, *MtCAD15*, *MtCAD17*, *MtCAD18*, *MtCAD19*, *MtCAD20*, *MtCAD25*, *MtCAD26*, *MtCAD28*, *MtCAD32*, *MtCAD33*, *MtCAD37*, *MtCAD41*, *MtCAD42*, *MtCAD45*, *MtCAD47*, *MtCAD48*, and *MtCAD49* showed up regulation. Lastly, *MtCAD1*, *MtCAD4*, *MtCAD6*, *MtCAD7*, *MtCAD10*, *MtCAD17*, *MtCAD19*, *MtCAD26*, *MtCAD27*, *MtCAD28*, *MtCAD32*, *MtCAD33*, *MtCAD37*, *MtCAD38*, *MtCAD42*, *MtCAD45*, *MtCAD47*, and *MtCAD48* showed during ABA treatment.

In *LjCAD*, cold stress led to treatment-induced increases in transcript levels in *LjCAD2*, *LjCAD4*, *LjCAD5*, *LjCAD6*, *LjCAD8*, *LjCAD9*, *LjCAD11*, *LjCAD13*, *LjCAD16*, *LjCAD17*, *LjCAD19*, *LjCAD22*, *LjCAD23*, *LjCAD28*, *LjCAD29*, *LjCAD30*, *LjCAD31*, *LjCAD33*, and *LjCAD35*. Heat stress induced higher expression in *LjCAD1*, *LjCAD4*, *LjCAD6*, *LjCAD7*, *LjCAD8*, *LjCAD9*, *LjCAD11*, *LjCAD19*, *LjCAD20*, *LjCAD24*, *LjCAD29*, *LjCAD32*, and *LjCAD34*. Under drought stress, *LjCAD2*, *LjCAD4*, *LjCAD5*, *LjCAD6*, *LjCAD7*, *LjCAD8*, *LjCAD9*, *LjCAD10*, *LjCAD11*, *LjCAD13*, *LjCAD15*, *LjCAD16*, *LjCAD17*, *LjCAD19*, *LjCAD20*, *LjCAD21*, *LjCAD22*, *LjCAD23*, *LjCAD24*, *LjCAD25*, *LjCAD27*, *LjCAD28*, *LjCAD29*, *LjCAD30*, *LjCAD31*, *LjCAD32*, *LjCAD33*, *LjCAD34*, and *LjCAD35* showed treatment-induced increases. NaCl stress also induced higher transcript levels in *LjCAD1*, *LjCAD2*, *LjCAD3*, *LjCAD8*, *LjCAD9*, *LjCAD11*, *LjCAD13*, *LjCAD16*, *LjCAD27*, *LjCAD28*, *LjCAD29*, *LjCAD32*, *LjCAD33*, *LjCAD34*, and *LjCAD35*. These results indicated potential stress-responsive CAD genes but functional validation is further necessary to confirm their roles in stress tolerance.

## 4. Discussion

Both ADH_N domain and ADH_zinc_N domain are crucial for the overall structure and function of cinnamyl alcohol dehydrogenase [54]. The structural integrity is maintained as the ADH_N domain functions as the catalytic core, while the ADH_zinc_N domain contributes to metal ion stabilization [55]. ADH_N is a GroES-like domain and is potentially involved in structural stabilization [56]. Legumes are exposed to various abiotic stresses such as cold, heat, drought and salinity stress. Under these stress conditions, CAD genes are upregulated to maintain efficient catalytic activity [57]. The larger number of CADs in *M. truncatula* compared with *L. japonicus* suggests possible differences in environmental adaptation. The physiochemical properties of MtCAD and LjCAD proteins unveiled that the longer amino acid sequences observed in LjCAD proteins may contribute to greater structural complexity compared with MtCAD proteins. Higher number of acidic protein indicated the enzymatic activity [58]. The instability index analysis highlighted that a majority of CAD proteins in both species were stable (39 MtCAD and 31 LjCAD), with *M. truncatula* displaying a slightly higher proportion of stable proteins [59].

A significant expansion in CAD proteins revealed through the largest group C based on the evolutionary phylogenetic tree. This reflects adaptive evolution linked to secondary cell wall formation, stress responses, or symbiotic interactions, which are critical for legume biology [60, 61]. Meanwhile, the smaller groups E and F exhibited strong purifying selection. Notably, LjCAD had a closer evolutionary relationship with *A. thaliana* compared with *M. truncatula* due to its position in phylogenetic tree. Therefore, despite both being legume*s,* they had different structure, function and regulatory mechanisms [62]. The closer affinity of LjCAD with AtCAD is due to shared evolutionary pressures, gene retention patterns, or functional constraints [63, 64].

The identification of nine major conserved motifs suggested that these regions are essential for proper protein folding and functional activity. In contrast, the presence of only a single motif in MtCAD51 may indicate functional specialization or divergence [65, 66]. Further analysis of major ADH_N and ADH_zinc_N domains revealed their contribution in alcohol dehydrogenase-like catalytic activity and zinc ion binding [67, 68]. A notable finding of introns in 49 out of 51 *MtCAD* and 35 *LjCAD* indicated their role in gene regulation and alternative splicing. Ultimately, the conservation of domain and motif regulates lignin biosynthesis in legumes [69, 70].

The clustering of *MtCAD* mostly on chromosome 5 (15 genes) and *LjCAD* on chromosome 1 (10 genes) along with 3 *MtCAD* (*MtCAD49*, *MtCAD50*, and *MtCAD51*) on scaffolds and 1 *LjCAD* (*LjCAD35*) on contig showed the exact locations of *CADs*. Further, identification of 12 collinear pairs in *MtCAD* and 9 collinear pair in *LjCAD* provided strong evidence for the structural conservation [71, 72]. Synteny analysis between *MtCAD* and *LjCAD* genes with other species provided their evolutionary relationships. It confirmed the closer evolutionary relationship of *LjCAD* compared to *MtCAD*. Previously, comparative genomic studies have demonstrated that conserved syntenic relationships among gene families reflect shared ancestry and functional conservation during plant evolution, particularly for genes involved in lignin biosynthesis and secondary cell wall formation [73, 74].

Gene duplication plays a key role in the expansion of gene families and contributes to functional redundancy, diversification, and environmental adaptation [75]. In this study, 12 pairs of duplicated gene in *MtCAD* and 9 pairs in *LjCAD* revealed both segmental and tandem pairs. The broad divergence times (between 6.08 MYA and 77.81 MYA in *MtCAD* and between 8.03 MYA and 48.71 MYA in *LjCAD*) indicated continuous *CAD* duplication [76]. Selective pressure analysis based on the nonsynonymous-to-synonymous substitution ratio (Ka/Ks) found to be less than 1 indicating purifying selection [77, 78]. This pattern suggested that duplicated CAD genes may retain conserved biological functions and that deleterious mutations have been removed through evolutionary constraints.

Subcellular localization analysis suggested that most MtCAD and LjCAD proteins are predicted to localize in the cytoplasm. This observation is consistent with the known role of CAD enzymes in the final steps of monolignol biosynthesis which primarily occur in the cytosol before monolignols are transported to the cell wall for lignin polymerization [79]. A smaller proportion of proteins were predicted to localize to other cellular compartments such as the chloroplast where specialized biosynthetic processes and secondary metabolism occurred [80]. But, experimental validation would be further required to confirm the precise cellular localization of these CAD proteins.

Cis-acting regulatory elements in promoter regions play an important role in controlling gene expression under environmental and hormonal stimuli [81]. In this study, a large number of light-responsive elements were identified in both *MtCAD* and *LjCAD*, suggesting that CAD genes may be transcriptionally regulated by light signals. These are known to influence lignin biosynthesis and plant development. The presence of ABRE and ARE elements indicates possible regulation by abscisic acid signaling and anaerobic stress conditions [82]. Furthermore, the detection of MeJA-responsive motifs (TGACG-motif and CGTCA-motif) and wound-responsive elements such as WUN-motif suggests that CAD genes may participate in plant defense and stress adaptation pathways [83]. These promoter regions ensure that CAD gene expression could be modulated by multiple environmental cues and hormonal signals.

Gene Ontology annotation suggested that most CAD proteins in both species, *MtCAD* and *LjCAD*, are associated with oxidoreductase activity and metabolic processes which is consistent with the enzymatic role of CAD in catalyzing the reduction of cinnamaldehydes to their corresponding alcohols during monolignol biosynthesis [84]. This particular function is relevant with the phenylpropanoid metabolic pathway for production of lignin and other secondary metabolites in plants [85].

PPI has crucial role in species diversification and cellular activity regulation [86, 87]. It revealed interactions between MtCAD, LjCAD, and *Arabidopsis* proteins showing their involvement in primary and secondary metabolism. MtCAD exhibited interactions with AtCAD7, while LjCAD showed strong interactions with AtADH1.

KEGG analysis further supported the functional role by linking several CAD genes to the phenylpropanoid biosynthesis pathway as well as to broader secondary metabolic pathways [88, 89]. These pathways are known to contribute to plant structural integrity, stress tolerance, and defense responses. Based on previous study, CAD enzymes catalyze the final step of lignin monomer biosynthesis within the phenylpropanoid pathway [74]. Although additional metabolic pathways were also detected, these associations may reflect the broader metabolic context of oxidoreductase enzymes rather than direct involvement in those pathways [90, 91]. Therefore, the presence of CAD genes in phenylpropanoid and secondary metabolite biosynthesis pathways provided the most biologically relevant functional insight.

Transcriptional and post-transcriptional control mechanisms are associated with TFs [92]. Several TF families were predicted to be associated with CAD genes, including ERF, MYB, NAC, bZIP, and bHLH. These TF families have previously been reported to participate in lignin biosynthesis and secondary cell wall formation in plants. For example, MYB and NAC transcription factors are well-established regulators of lignin biosynthetic pathways, while ERF family members have also been implicated in stress-induced lignin accumulation [93, 94]. Therefore, the predicted TF-CAD regulatory network may reflect potential regulatory mechanisms controlling CAD expression.

MicroRNAs are important regulators of gene expression in plants and frequently control genes involved in development, stress responses, and secondary metabolism [95]. A larger number of miRNAs were predicted to target *MtCAD* genes (510 miRNAs targeting 49 genes) compared with *LjCAD* (21 miRNAs targeting 12 genes), suggesting potentially more complex post-transcriptional regulation in M. truncatula. Several miRNAs were predicted to regulate multiple CAD genes. This indicated that certain miRNAs may coordinate the regulation of lignin biosynthesis-related genes. Previous studies have shown that miRNAs can modulate stress-responsive pathways and secondary cell wall formation, processes closely associated with lignin metabolism [96]. However, the regulatory relationships identified require experimental validation.

Plants use tissue-specific gene expression as their fundamental biological mechanism to optimize their responses to stress through activating stress-responsive genes in particular cells or tissues [97, 98]. Tissue-specific expression patterns provided understanding the roles in plant growth, development, and organ differentiation [99]. For instance, the high expression of *MtCAD25* in buds and *LjCAD23* in immature flowers suggested their involvement in early reproductive development, while the highest expression of *MtCAD3* and *MtCAD9* in roots indicated functions related to nutrient uptake and stress adaptation. Expression analysis of *PoptrCAD* genes revealed that they expressed in leaves, petioles, bark, and xylem, although their expression levels varied among these tissues. For instance, PoptrCAD7 showed relatively higher expression in leaves and petioles but exhibited very low expression in bark and xylem [100]. Some genes including *MtCAD42* and *MtCAD47* showed relatively high expression across multiple tissues suggesting broader metabolic or structural functions.

Under cold stress, 12 *MtCAD* and 13 *LjCAD* genes exhibited increased transcript levels and suggested their potential involvement in cold-responsive metabolic pathways, cellular protection mechanisms, and regulatory networks. In pepper, CaCAD1 expression increased under low temperature and it contributed to lignin biosynthesis and cold stress response [101].

Among these, *MtCAD27* and *LjCAD11* showed the highest transcript accumulation, indicating particularly strong cold-responsive behavior. During drought stress, 29 *MtCAD* and 30 *LjCAD* genes showed increased expression, with *MtCAD10* and *LjCAD16* displaying the largest treatment-induced regulation. In melon, drought stress significantly induced the expression of CmCAD genes and promoted lignin biosynthesis in stems [102]. Increased expression of CAD genes under drought has also been associated with enhanced lignin accumulation and improved stress adaptation in several plant species [103]. Under freezing stress, among 24 increased level of expression of *MtCAD* genes, *MtCAD10* exhibited the highest transcript levels. Similarly, under salt stress, *MtCAD11* showed the most significant increase while ABA treatment induced maximal expression in *MtCAD28*. In *LjCAD*, *LjCAD11* showed the highest treatment-induced expression under both heat and NaCl stress. These findings highlight stress-responsive candidates but functional validation is required to confirm their roles in stress tolerance.

## 5. Conclusion

The comparative genome-wide study of 51 *MtCAD* and 35 *LjCAD* provided valuable insights into their role under different abiotic stresses. All the MtCAD and LjCAD proteins contained ADH_N domain. Evolutionary analysis with *A. thaliana* indicated a closer association of *LjCAD* genes with *AtCAD* and thus it reflected shared evolutionary pressures. The subcellular localization indicated that majority of *MtCAD* and *LjCAD* were found in chloroplast. Furthermore, *MtCAD* and *LjCAD* genes were unevenly distributed across eight and six chromosomes, respectively, exhibiting distinct exon-intron structures and gene lengths. Purifying selection was observed across all *MtCAD* and *LjCAD* genes, consistent with their conserved evolutionary relationships. Functional annotations revealed the role of *MtCAD* in oxidation-reduction process along with the role of *LjCAD* in oxidoreductase activity. ERF was the largest TF found in both *MtCAD* and *LjCAD.* The expression revealed the increased transcript level in *MtCAD1*, *MtCAD3*, *MtCAD9*, *MtCAD15*, *MtCAD23*, *MtCAD27*, and *MtCAD47* under cold, drought and freezing stress and *LjCAD6*, *LjCAD8*, and *LjCAD11* under cold, drought and heat stress. These patterns indicated that these genes are potential stress-responsive candidates under multiple abiotic conditions. Such findings will provide a basis for future studies and breeding programs for developing plants with improved stress resilience.

## Supporting information

S1 File S1 Data. Phylogenetic tree sequences of CAD proteins. S2 Data. 2000 bp sequences of MtCAD and LjCAD gene families. S3 Data. Peptide sequences of MtCAD and LjCAD. S4 Data. CDS of MtCAD and LjCAD. S5 Data. Genomic sequences of MtCAD and LjCAD. S6 Data. Exon and intron counts of MtCAD and LjCAD.S7 Data. KaKs ratio of MtCAD and LjCAD. S8 Data. CARE of MtCAD and LjCAD. S9 Data. GO of MtCAD and LjCAD. S10 Data. PPI of MtCAD and LjCAD. S11 Data. PPI of MtCAD and LjCAD. S12 Data. MicroRNA of MtCAD and LjCAD. S13 Data. Tissue specific expression of MtCAD and LjCAD. S14 Data. Abiotic stress expression of MtCAD and LjCAD.
(ZIP)

S1 Fig Phylogenetic distribution of the CAD. (TIFF)

S2 Fig A. Different colors indicate individual motifs for MtCAD. B. Specific-colored box aligned on the right side of the figure shows 10 different motifs. Different colors indicate individual motifs for LjCAD.
(TIFF)

S3 Fig A. The purple box illustrates the conserved domain, whereas the blue box illustrates entire protein of respective MtCAD. B. The blue box illustrates the conserved domain, whereas the green box illustrates entire protein of respective MtCAD.
(TIFF)

S4 Fig A. The black lines represent introns, blue represents exons, and green lines represent upstream/downstream region. B. The black lines represent introns, blue represents exons, and deep pink lines represent upstream/downstream region.
(TIFF)

S5 Fig A. Orange color rectangles represent chromosomes 1–8. Scf represents scaffold chromosome. B. The collinear relationship of the *LjCAD*. Green rectangles represent chromosomes 1–6. Contig represents contiguous chromosome. The dark blue lines represent collinear relations between them.
(TIFF)

S6 Fig A. The Ka/Ks of *MtCAD* represents the ratio of Ka to Ks, with divergence time (measured in million years ago, MYA). B. The Ka/Ks of *LjCAD* represents the ratio of Ka to Ks, with divergence time (measured in million years ago, MYA). The color bar represents the range of value.
(TIFF)

S7 Fig A. The absence and presence of the respective *MtCAD* genes in various organelles are shown. B. The percentage of *MtCAD* gene location across various cellular organelles is represented by a deep purple bar diagram. C. The absence and presence of the respective *LjCAD* genes in various organelles are shown. D. The percentage of *LjCAD* gene location across various cellular organelles is represented by a deep blue diagram.
(TIFF)

S8 Fig A. The MtCAD protein-protein interaction displayed at network nodes with the proteins in nodes, and the line colors indicate different data sources. B. The LjCAD protein- protein interaction displayed at network nodes with the proteins in nodes, and the line colors indicate different data sources.
(TIFF)

S9 Fig A. The heat map represents transcription factors (TFs) in *MtCAD*. The orange box on the right side of the heat map indicates the presence of TFs in genes. B. The heat map represents transcription factors (TFs) in *LjCAD*. The green box on the right side of the heat map indicates the presence of TFs in genes.
(TIFF)

S10 Fig A. This figure shows the interactions between different TFs and *MtCAD* gene. On the right side of the figure, the color representation is shown. B. This figure shows the interactions between different TFs and *LjCAD* gene. On the right side of the figure, the color representation is shown.
(TIFF)

S11 Fig A. The purple round rectangle represents the exons of the *MtCAD* gene, straight black line represents intron and red color small round rectangle represents microRNA (miRNA). B. The blue round rectangle represents the exons of the *LjCAD* gene, straight black line represents intron and red color small round rectangle represents microRNA (miRNA).
(TIFF)
