# Supplementary material for: Comparative genome-wide analysis of CAD (Cinnamyl Alcohol Dehydrogenase) gene family in Medicago truncatula and Lotus japonicus and their expression profiles in response to various abiotic abiotic stresses
Source: PLoS One. 2026 Jul 21;21(7):e0353726. doi: 10.1371/journal.pone.0353726 (PMC13387551; doi:10.1371/journal.pone.0353726)
Supplement: S1 File — Phylogenetic tree sequences of CAD proteins. S2 Data. 2000 bp sequences of MtCAD and LjCAD gene families. S3 Data. Peptide sequences of MtCAD and LjCAD. S4 Data. CDS of MtCAD and LjCAD. S5 Data. Genomic sequences of MtCAD and LjCAD. S6 Data. Exon and intron counts of MtCAD and LjCAD.S7 Data. KaKs ratio of MtCAD and LjCAD. S8 Data. CARE of MtCAD and LjCAD. S9 Data. GO of MtCAD and LjCAD. S10 Data. PPI of MtCAD and LjCAD. S11 Data. PPI of MtCAD and LjCAD. S12 Data. MicroRNA of MtCAD and LjCAD. S13 Data. Tissue specific expression of MtCAD and LjCAD. S14 Data. Abiotic stress expression of MtCAD and LjCAD. (ZIP) [file pone.0353726.s001.zip › Supporting Information/Supplementary data/S6 Data. Exon and intron counts of MtCAD and LjCAD.docx]

**S6 A Data. *MtCAD* exon and intron counts:**

| Gene name | Intron | Exon |
| --- | --- | --- |
| MtCAD1 | 4 | 5 |
| MtCAD2 | 8 | 9 |
| MtCAD3 | 4 | 5 |
| MtCAD4 | 6 | 7 |
| MtCAD5 | 5 | 6 |
| MtCAD6 | 5 | 6 |
| MtCAD7 | 7 | 8 |
| MtCAD8 | 9 | 10 |
| MtCAD9 | 9 | 10 |
| MtCAD10 | 9 | 10 |
| MtCAD11 | 9 | 10 |
| MtCAD12 | 5 | 6 |
| MtCAD13 | 9 | 10 |
| MtCAD14 | 4 | 5 |
| MtCAD15 | 5 | 6 |
| MtCAD16 | 17 | 18 |
| MtCAD17 | 18 | 19 |
| MtCAD18 | 4 | 5 |
| MtCAD19 | 4 | 5 |
| MtCAD20 | 4 | 5 |
| MtCAD21 | 4 | 5 |
| MtCAD22 | 4 | 5 |
| MtCAD23 | 4 | 5 |
| MtCAD24 | 3 | 4 |
| MtCAD25 | 4 | 5 |
| MtCAD26 | 4 | 5 |
| MtCAD27 | 4 | 5 |
| MtCAD28 | 4 | 5 |
| MtCAD29 | 1 | 2 |
| MtCAD30 | 3 | 4 |
| MtCAD31 | 3 | 4 |
| MtCAD32 | 8 | 9 |
| MtCAD33 | 8 | 9 |
| MtCAD34 | 3 | 4 |
| MtCAD35 | 3 | 4 |
| MtCAD36 | 9 | 10 |
| MtCAD37 | 5 | 6 |
| MtCAD38 | 5 | 6 |
| MtCAD39 | 4 | 5 |
| MtCAD40 | 9 | 10 |
| MtCAD41 | 9 | 10 |
| MtCAD42 | 7 | 8 |
| MtCAD43 | 2 | 3 |
| MtCAD44 | 2 | 3 |
| MtCAD45 | 3 | 4 |
| MtCAD46 | 4 | 5 |
| MtCAD47 | 10 | 11 |
| MtCAD48 | 7 | 8 |
| MtCAD49 | 7 | 8 |
| MtCAD50 | 0 | 1 |
| MtCAD51 | 0 | 1 |

**S6 A Data. *LjCAD* exon and intron counts:**

| Gene name | Intron | Exon |
| --- | --- | --- |
| LjCAD1 | 5 | 6 |
| LjCAD2 | 8 | 9 |
| LjCAD3 | 7 | 8 |
| LjCAD4 | 9 | 10 |
| LjCAD5 | 9 | 10 |
| LjCAD6 | 8 | 9 |
| LjCAD7 | 3 | 4 |
| LjCAD8 | 9 | 10 |
| LjCAD9 | 9 | 10 |
| LjCAD10 | 6 | 7 |
| LjCAD11 | 4 | 5 |
| LjCAD12 | 3 | 4 |
| LjCAD13 | 5 | 6 |
| LjCAD14 | 4 | 5 |
| LjCAD15 | 4 | 5 |
| LjCAD16 | 3 | 4 |
| LjCAD17 | 9 | 10 |
| LjCAD18 | 3 | 4 |
| LjCAD19 | 7 | 8 |
| LjCAD20 | 11 | 12 |
| LjCAD21 | 9 | 10 |
| LjCAD22 | 4 | 5 |
| LjCAD23 | 7 | 8 |
| LjCAD24 | 5 | 6 |
| LjCAD25 | 5 | 6 |
| LjCAD26 | 17 | 18 |
| LjCAD27 | 5 | 6 |
| LjCAD28 | 8 | 9 |
| LjCAD29 | 4 | 5 |
| LjCAD30 | 9 | 10 |
| LjCAD31 | 9 | 10 |
| LjCAD32 | 5 | 6 |
| LjCAD33 | 9 | 10 |
| LjCAD34 | 5 | 6 |
| LjCAD35 | 9 | 10 |
