# Supplementary figures and images for: Comparative genome-wide analysis of CAD (Cinnamyl Alcohol Dehydrogenase) gene family in Medicago truncatula and Lotus japonicus and their expression profiles in response to various abiotic abiotic stresses
Source: PLoS One. 2026 Jul 21;21(7):e0353726. doi: 10.1371/journal.pone.0353726 (PMC13387551; doi:10.1371/journal.pone.0353726)

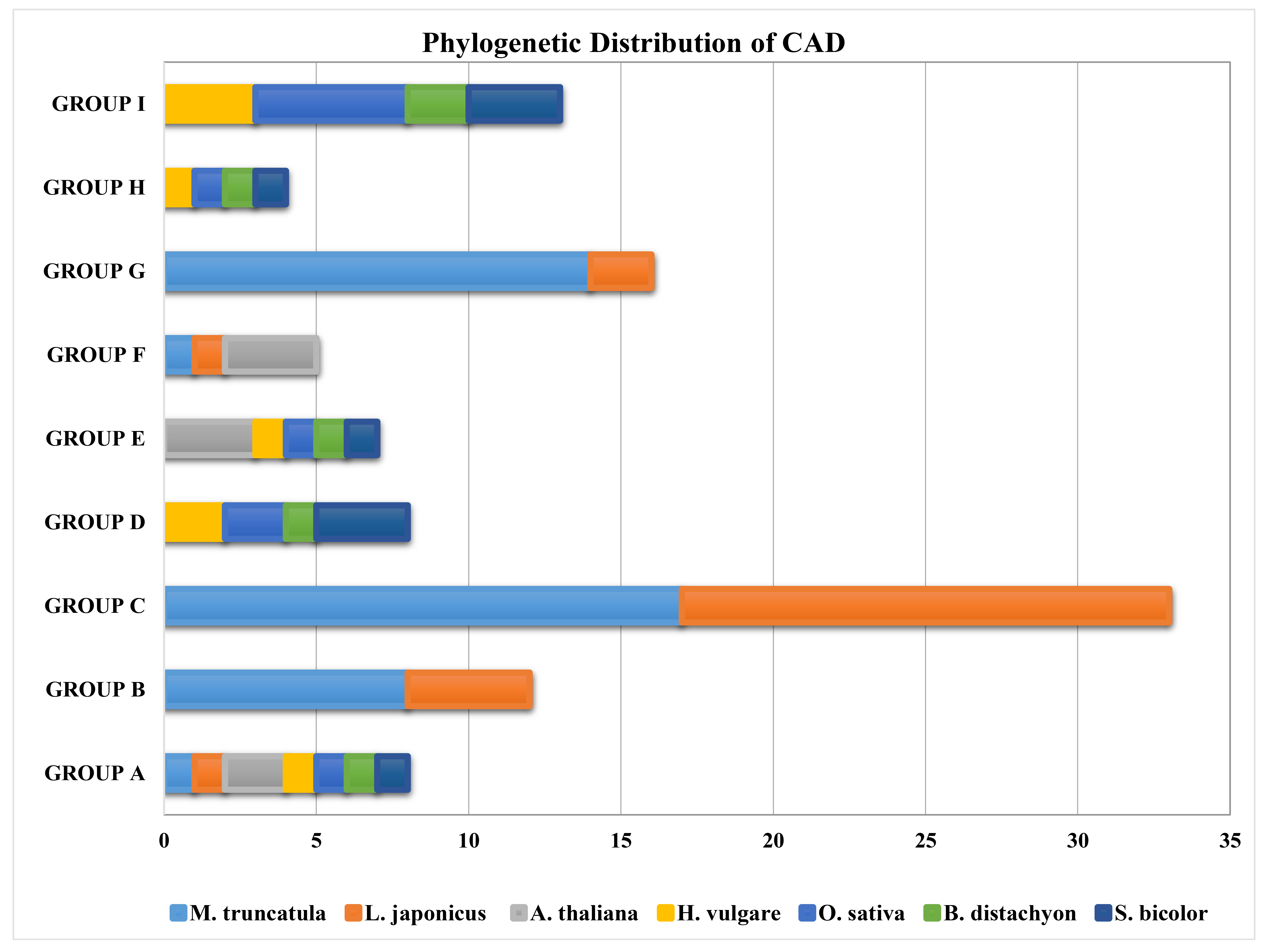

Supplement: S1 Fig — (TIFF) [file pone.0353726.s002.tiff]

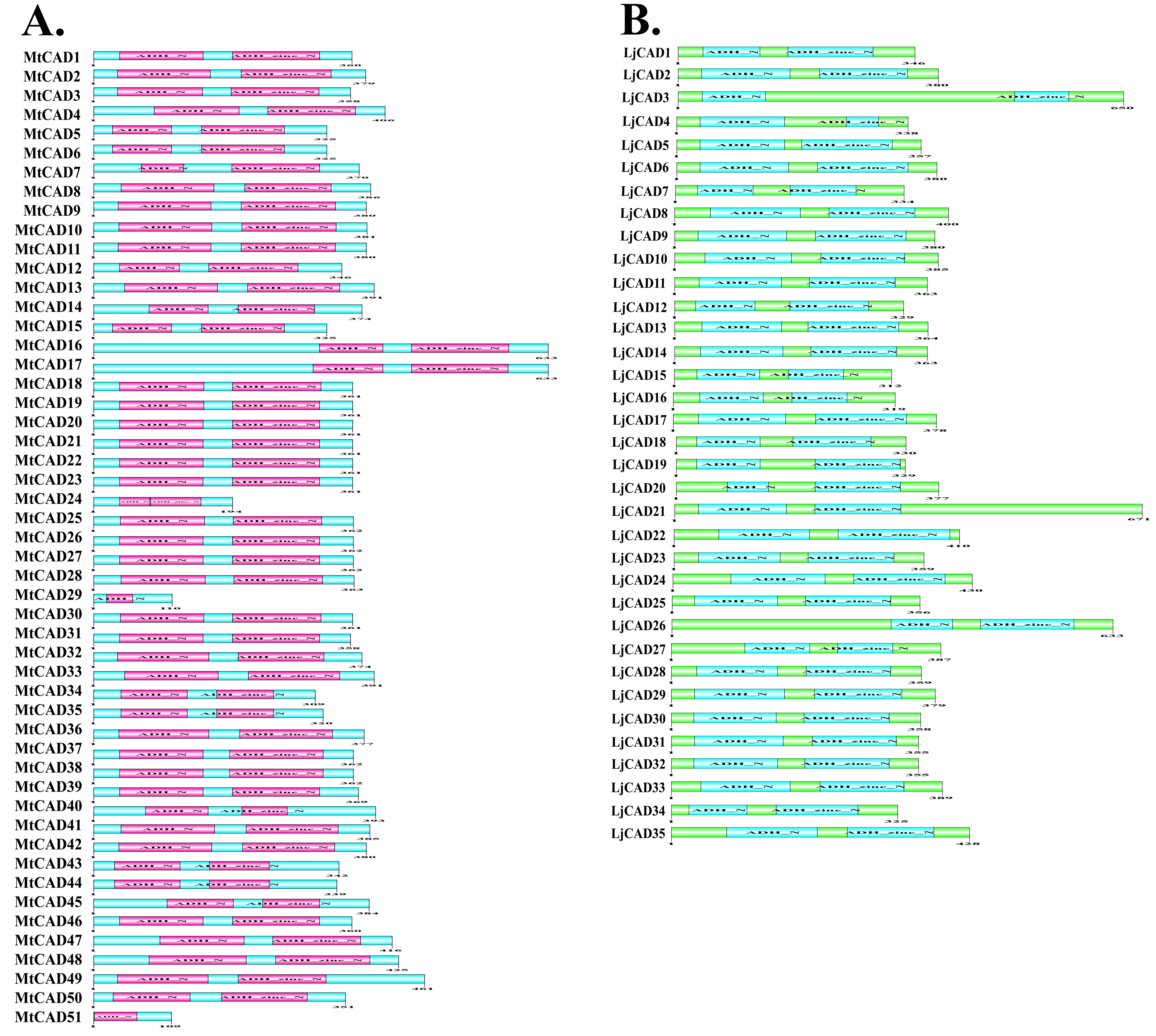

Supplement: S3 Fig — B. The blue box illustrates the conserved domain, whereas the green box illustrates entire protein of respective MtCAD. (TIFF) [file pone.0353726.s004.tiff]

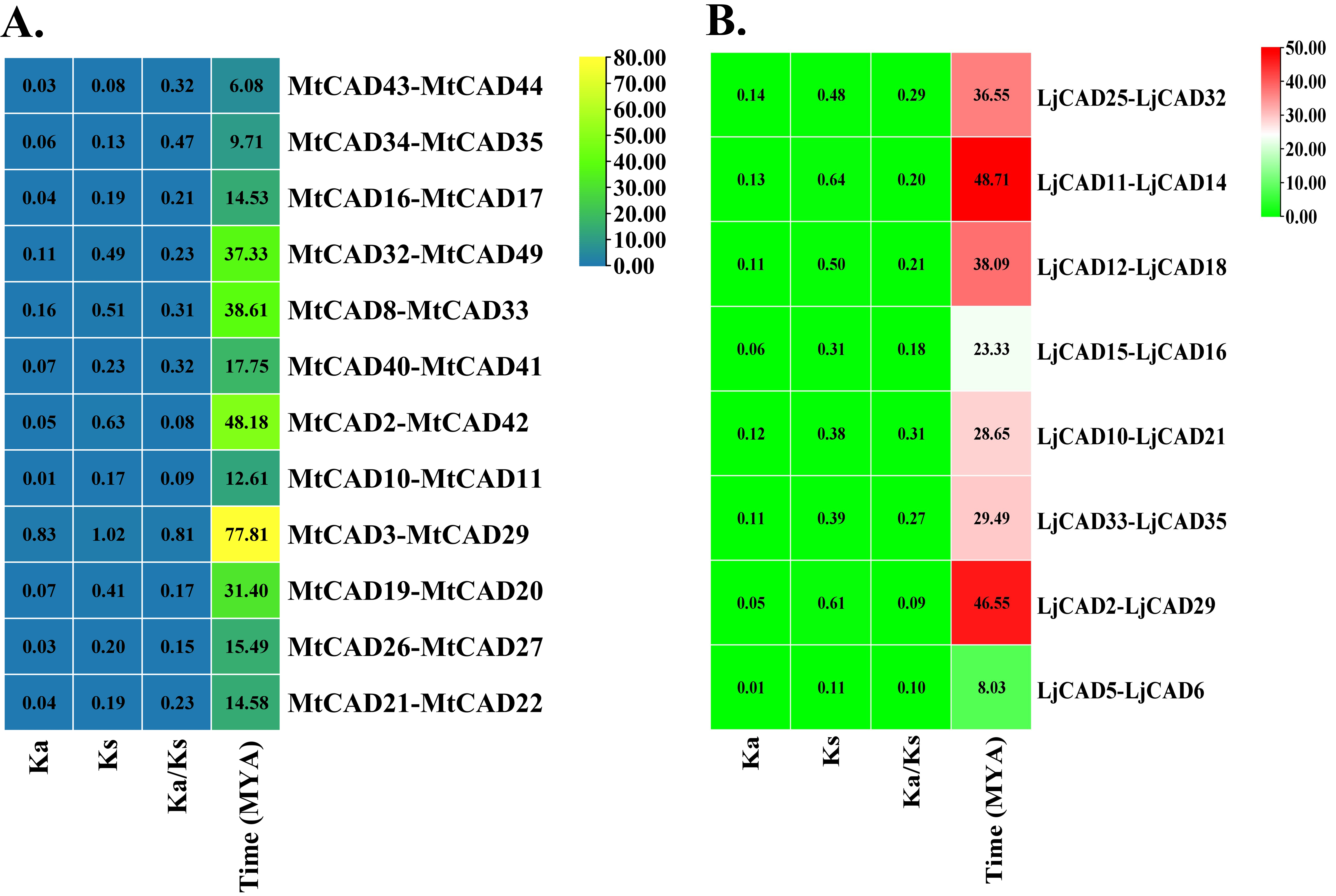

Supplement: S6 Fig — B. The Ka/Ks of LjCAD represents the ratio of Ka to Ks, with divergence time (measured in million years ago, MYA). The color bar represents the range of value. (TIFF) [file pone.0353726.s007.tiff]

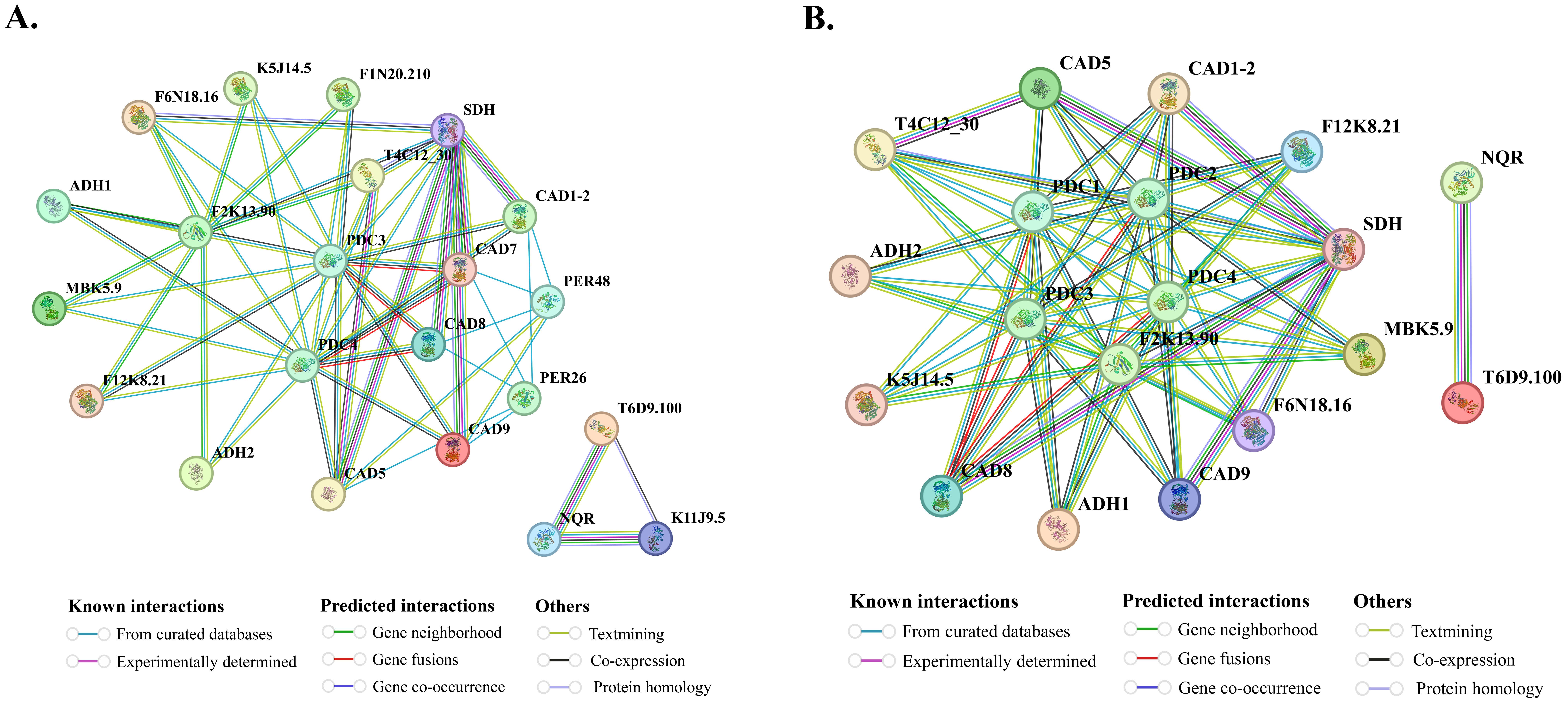

Supplement: S8 Fig — B. The LjCAD protein- protein interaction displayed at network nodes with the proteins in nodes, and the line colors indicate different data sources. (TIFF) [file pone.0353726.s009.tiff]

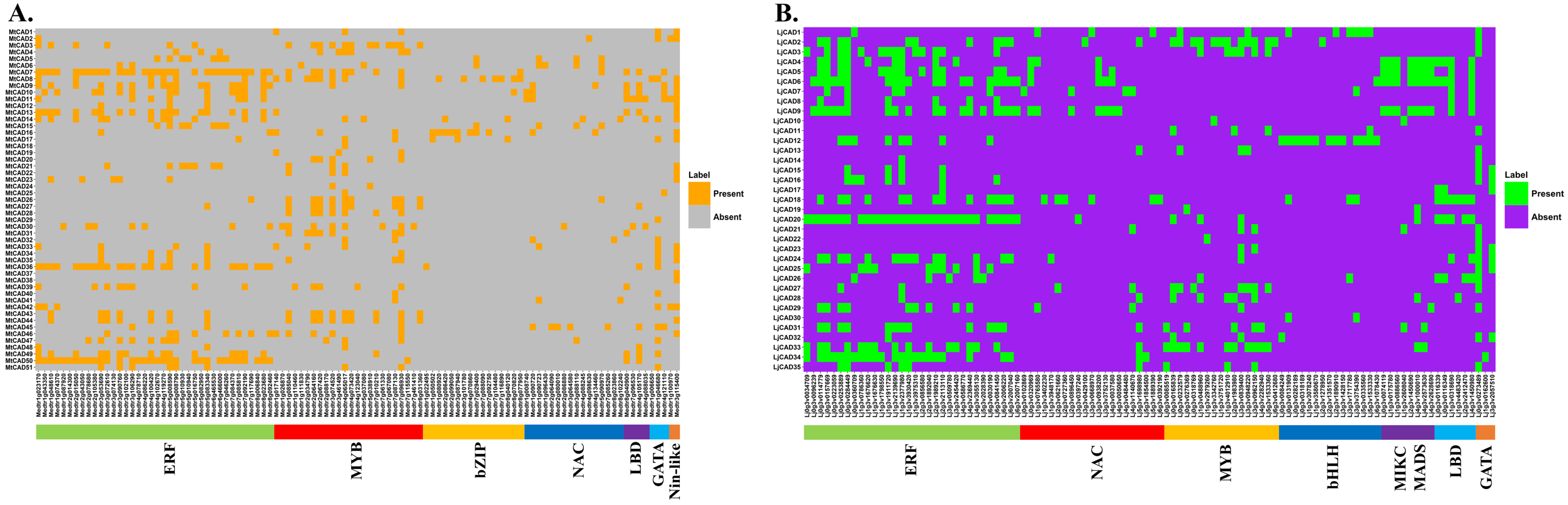

Supplement: S9 Fig — B. The heat map represents transcription factors (TFs) in LjCAD. The green box on the right side of the heat map indicates the presence of TFs in genes. (TIFF) [file pone.0353726.s010.tiff]

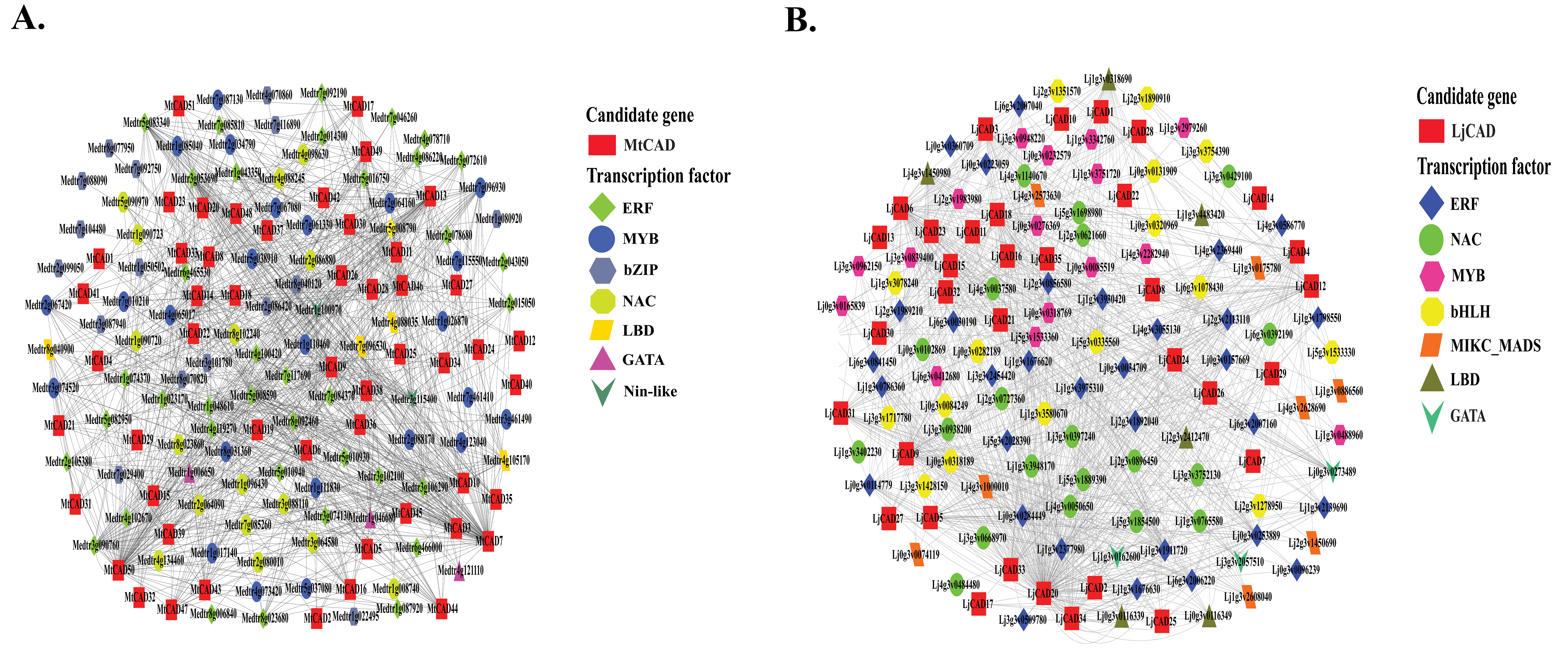

Supplement: S10 Fig — This figure shows the interactions between different TFs and LjCAD gene. On the right side of the figure, the color representation is shown. (TIFF) [file pone.0353726.s011.tiff]

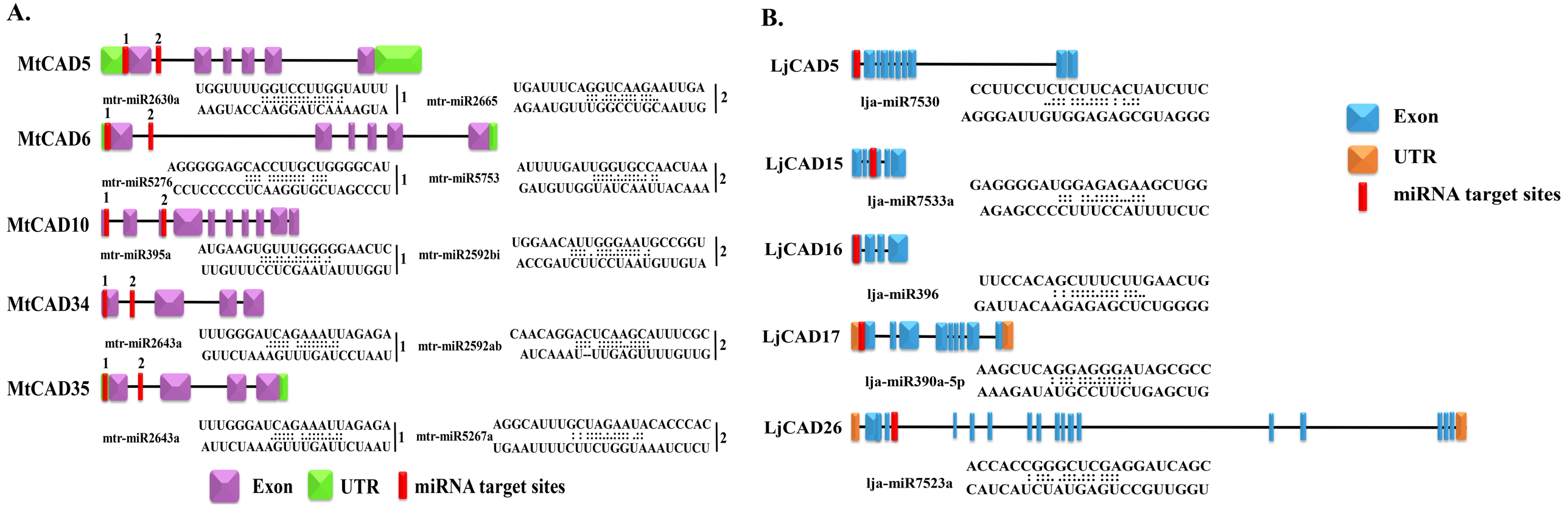

Supplement: S11 Fig — B. The blue round rectangle represents the exons of the LjCAD gene, straight black line represents intron and red color small round rectangle represents microRNA (miRNA). (TIFF) [file pone.0353726.s012.tiff]
